# Circulating Tumor DNA in Resected Colorectal Liver Metastases: From Minimal Residual Disease Detection to Treatment Guidance—A Systematic Review

**DOI:** 10.3390/cancers18203304

**Published:** 2026-10-14

**Authors:** Melina Yerolatsite, Nanteznta Torounidou, Loizos Hadjigeorgiou, Anna-Lea Amylidi, Evangeli Lampri, Georgios D. Lianos, Angeliki Magklara, George Zarkavelis

**Affiliations:** 1 Department of Medical Oncology, School of Health Sciences, Faculty of Medicine, University of Ioannina, 45110 Ioannina, Greece; annalea.ami@gmail.com (A.-L.A.); g.zarkavelis@uoi.gr (G.Z.); 2 Department of Medical Oncology, University Hospital of Ioannina, 45500 Ioannina, Greece; nadia.torou@gmail.com; 3 Department of General Surgery, University Hospital of Ioannina, 45500 Ioannina, Greece; loizoshadjigeorgiou@gmail.com (L.H.); glianos@uoi.gr (G.D.L.); 4 Department of Pathology, University of Ioannina, 45110 Ioannina, Greece; elampri@uoi.gr; 5 Department of Clinical Chemistry, Faculty of Medicine, School of Health Sciences, University of Ioannina, 45110 Ioannina, Greece; magklara@uoi.gr; 6 Biomedical Research Institute-Foundation for Research and Technology, 45110 Ioannina, Greece; 7 Society for Study of Clonal Heterogeneity of Neoplasia (EMEKEN), 45444 Ioannina, Greece

**Keywords:** colorectal neoplasms, liver neoplasms, circulating tumor DNA, residual, neoplasm recurrence, local, prognosis, biomarkers, tumor, liquid biopsy

## Abstract

**Background**: Circulating tumor DNA (ctDNA) has emerged as a potential marker of minimal residual disease (MRD) after resection of colorectal liver metastases (CRLM), but its prognostic value and possible role in guiding postoperative management remain uncertain. This systematic review evaluated the available evidence on ctDNA for predicting recurrence and survival after CRLM resection, with particular attention to perioperative and serial ctDNA measurements and their potential value in guiding adjuvant treatment. **Methods**: PubMed/MEDLINE, Scopus, and the Cochrane Library were searched from inception to 21 July 2026 for studies evaluating ctDNA in patients undergoing resection of CRLM. Prospective and retrospective cohort studies and relevant randomized trial sub-studies were eligible. The primary outcome was the association between ctDNA status and recurrence, while secondary outcomes included recurrence-free survival (RFS), disease-free survival (DFS), overall survival (OS), serial ctDNA dynamics, and the potential predictive value of ctDNA for adjuvant chemotherapy. Risk of bias was assessed using the QUIPS tool. Findings were synthesized narratively, with an additional exploratory random-effects meta-analysis of early postoperative tissue-informed ctDNA positivity and RFS/DFS. Unadjusted and adjusted HRs were pooled separately. **Results**: Twenty-eight studies published between 2018 and 2026 were included, representing 2079 unique patients after accounting for overlapping cohorts. In six studies, postoperative ctDNA positivity was associated with poorer RFS/DFS (pooled unadjusted HR 5.15, 95% CI 3.65–7.27; I^2^ = 0%). Five studies provided adjusted estimates (pooled HR 4.95, 95% CI 2.45–10.01; I^2^ = 60.5%). Serial monitoring provided additional prognostic information: persistent ctDNA positivity or conversion from negative to positive during follow-up was associated with poor outcomes, whereas ctDNA clearance was generally associated with a lower risk of recurrence. ctDNA could precede radiological recurrence by approximately 2.5 to >20 months, although detection was less reliable in patients with small-volume pulmonary recurrence. In contrast, the prognostic value of preoperative ctDNA was less consistent and appeared to depend on assay methodology, quantitative ctDNA burden, and previous systemic treatment. Observational studies reported associations between postoperative MRD status and outcomes following adjuvant chemotherapy, but non-randomized treatment allocation and potential time-related bias limit conclusions about treatment benefit. Considerable variation in assay platforms and postoperative sampling schedules remained across studies. **Conclusions**: Postoperative ctDNA positivity is associated with poorer recurrence-related outcomes after CRLM resection. However, the moderate-to-high risk of bias and differences between studies limit confidence in the evidence. The predictive value of ctDNA for adjuvant chemotherapy benefit remains unproven. Prospective randomized trials are needed to determine whether ctDNA-guided management improves patient outcomes.

## Simple Summary

Surgery can remove colorectal cancer that has spread to the liver, but the cancer often returns. Blood tests that look for DNA released by cancer cells may help identify patients who are more likely to develop a recurrence. We reviewed 28 studies of these tests in patients undergoing surgery for colorectal liver metastases. Patients with tumor DNA detected in their blood after surgery were more likely to have their cancer return. In some cases, repeated blood tests detected signs of recurrence before scans did. However, a negative test did not rule out recurrence, especially when small lung metastases were present. The studies differed in their methods and had limitations that made the findings less certain. Further trials are needed to determine whether using these blood tests to choose treatment or change follow-up helps patients live longer or reduces recurrence.

## 1. Introduction

Colorectal cancer (CRC) is the third most commonly diagnosed cancer, accounting for approximately 10% of all cancer cases and it is the second leading cause of cancer-related deaths worldwide [1]. The prognosis of colorectal cancer largely depends on disease stage at diagnosis. Despite potentially curative surgery, with or without adjuvant chemotherapy, approximately 30–50% of patients will eventually develop recurrent disease, which remains the leading cause of CRC-related mortality [2]. The liver is the most frequent site of metastasis, and colorectal liver metastases (CRLM) remain the leading cause of disease-related death in patients with advanced CRC [3,4].

In addition, adjuvant chemotherapy is well established as the standard of care for stage III (node-positive) colorectal cancer because of its proven survival benefit. In patients with stage I and II disease, however, treatment decisions are more complex. While only a subset of these patients will develop recurrence, predicting who is truly at high risk remains a challenge. Conventional clinicopathological factors, such as T4 disease, bowel perforation or obstruction, inadequate lymph node sampling, poor histological differentiation, and lymphovascular invasion, are helpful but have limited accuracy. Yet, these clinicopathological markers often lack the precision we need in daily practice [5,6].

Current follow-up of patients with colorectal cancer mainly relies on carcinoembryonic antigen (CEA) measurements and CT imaging. However, CEA has limited sensitivity and specificity, while CT may fail to detect small lesions and can produce false-positive findings, in addition to exposing patients to repeated radiation. These limitations make reliable monitoring of disease burden and early detection of recurrence challenging [7,8,9]. Similar issues arise when evaluating responses to treatment. Although CT-based Response Evaluation Criteria in Solid Tumors (RECIST) remain the standard method for response assessment, their use is limited by inter- and intra- observer variability and by the fact that some patients do not have measurable disease on imaging. Therefore, more sensitive and less invasive methods that can be used repeatedly over time may improve both surveillance and assessment of treatment response [10,11]. As a result, there is still a need for more reliable biomarkers that can improve risk stratification, guide treatment decisions, and enable the early detection of recurrent or metastatic disease during follow-up [11].

Advances in molecular diagnostics and a deeper understanding of the molecular pathways involved in colorectal carcinogenesis have greatly improved opportunities for early cancer detection, disease monitoring, and personalized treatment strategies. Among the emerging biomarkers, cell-free DNA (cfDNA) has attracted considerable interest because it can be detected in body fluids such as blood and reflects ongoing cellular turnover. First described in 1948, cfDNA is released primarily through apoptosis and necrosis, and its circulating levels are consistently higher in patients with cancer than in healthy individuals. Consequently, cfDNA has been extensively investigated as a non-invasive biomarker across a wide range of malignancies [12,13,14,15].

In patients with cancer, a proportion of circulating cfDNA originates directly from tumor cells and is referred to as circulating tumor DNA (ctDNA). Because ctDNA carries the same genetic alterations as the primary tumor, it provides a highly specific source of molecular information that can be used for disease detection, treatment monitoring, and assessment of residual disease. Current ctDNA assays commonly rely on the identification of tumor-specific somatic mutations, which are first characterized in tumor tissue and subsequently tracked in plasma samples [16,17,18].

Growing evidence supports the use of ctDNA as a valuable biomarker for minimal residual disease (MRD) detection, surveillance for recurrence, and evaluation of treatment response in colorectal cancer. Various analytical approaches have been developed for ctDNA analysis, with digital droplet PCR (ddPCR) being one of the most widely adopted because of its high analytical sensitivity. However, ddPCR assays targeting predefined tumor-specific alterations, such as KRAS mutations, are applicable only to patients whose tumors harbor the respective alterations. Broader tumor-informed and mutation-independent approaches may increase patient coverage, although their analytical performance and degree of clinical validation vary. Consequently, the prognostic role of ctDNA in MRD detection and disease monitoring after resection of colorectal liver metastases remains incompletely defined [16,17,18,19,20,21,22,23,24].

The aim of this systematic review was to summarize the available evidence on the role of ctDNA in patients undergoing resection of colorectal liver metastases, focusing on its value for detecting minimal residual disease, predicting recurrence and survival, monitoring disease after surgery, and its potential role in guiding adjuvant treatment.

## 2. Methods

### 2.1. Literature Review

This systematic review was conducted and reported in accordance with the Preferred Reporting Items for Systematic Reviews and Meta-Analyses (PRISMA 2020) statement. The review protocol was prospectively registered in PROSPERO (registration number: CRD420261484297) and is available through the PROSPERO database.

### 2.2. Search Strategy

A systematic literature search was performed in PubMed/MEDLINE, Scopus, and the Cochrane Library from database inception through 21 July 2026. The search combined terms related to colorectal cancer, colorectal liver metastases, ctDNA, cell-free DNA (cfDNA), liquid biopsy, and MRD. The complete search strategy for each database is provided in the Supplementary Data S1. To ensure that no relevant studies were missed, the reference lists of all included articles and relevant review papers were also screened manually. The complete search strategy for each database is provided in the Supplementary Data S1.

### 2.3. Study Selection

Studies investigating the prognostic or clinical role of ctDNA in patients undergoing resection of colorectal liver metastases were eligible for inclusion. Prospective and retrospective cohort studies, as well as randomized clinical trials reporting ctDNA-related outcomes, were considered.

Studies were excluded if they did not include patients with colorectal liver metastases, did not report ctDNA findings separately, were reviews, editorials, letters, conference abstracts, or preclinical studies, or if the full text was unavailable.

**Figure 1:**
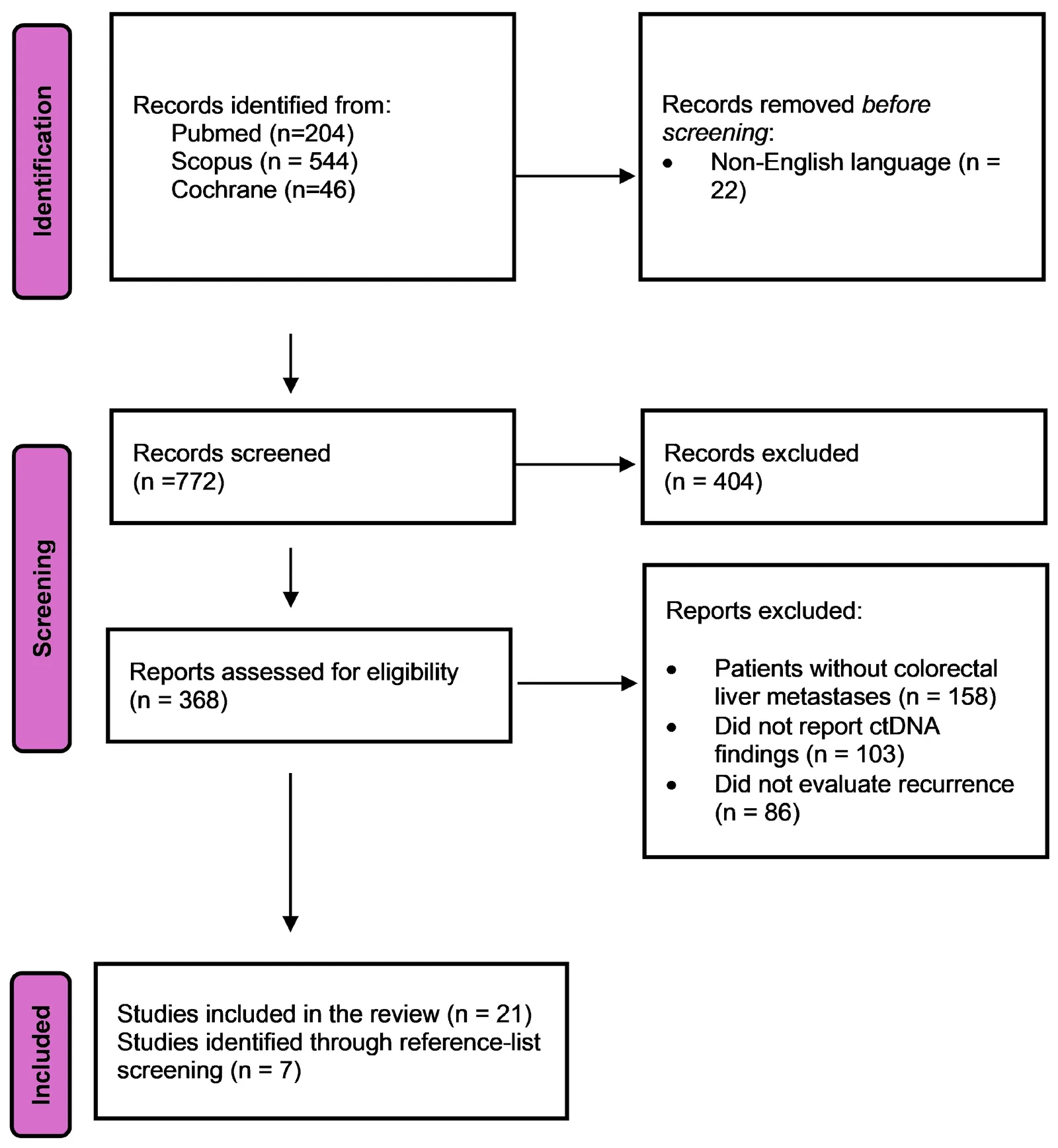
PRISMA flow diagram of the study selection process.

Titles, abstracts, and full-text articles were screened independently by two reviewers (Μ.Υ. and Ν.T.). Any disagreements were resolved through discussion, with consultation of a senior author (G.Z.) when required. The PRISMA flow diagram of the study selection process is presented in Figure 1.

### 2.4. Data Extraction

The following data were extracted from each study: first author, year of publication, country, study design, sample size, patient demographics (age and sex), number and extent of liver metastases, treatment details, ctDNA detection method, timing of blood sampling, ctDNA findings, diagnostic performance (sensitivity and specificity, where reported), recurrence, survival outcomes, and the main study limitations and conclusions.

### 2.5. Risk of Bias Assessment

The methodological quality of the included prognostic studies was independently assessed by two reviewers using the Quality in Prognosis Studies (QUIPS) tool. Six domains were evaluated: study participation, study attrition, prognostic factor measurement, outcome measurement, study confounding, and statistical analysis and reporting. Each domain was rated as having a low, moderate, or high risk of bias according to the QUIPS guidance. Overall risk of bias was classified as high when at least one domain was rated as high, moderate when at least one domain was rated as moderate and none as high, and low when all six domains were rated as low. Judgments referred to the prognostic contrast specified for each study, as different outcomes and sampling time points could have different sources of bias. During revision, we assessed the certainty of evidence for the association between early postoperative ctDNA positivity and RFS/DFS using GRADE guidance for prognostic factors [25]. Observational evidence started at high certainty and was assessed for risk of bias, inconsistency, indirectness, imprecision and publication bias. Unadjusted and adjusted estimates were assessed separately. The assessment addressed the presence of an adverse prognostic association (HR > 1), rather than its precise magnitude or the clinical benefit of ctDNA-guided management. QUIPS judgments informed the risk-of-bias assessment, and no upgrading was applied.

### 2.6. Outcomes

The primary outcome was the association between ctDNA status and recurrence after resection of colorectal liver metastases. Secondary outcomes included recurrence-free survival (RFS), disease-free survival, overall survival (OS), the value of serial postoperative ctDNA monitoring, and the potential role of ctDNA in selecting patients for adjuvant chemotherapy.

### 2.7. Data Synthesis

The overall evidence was synthesized narratively because of differences in study design, patient populations, ctDNA assays, sampling schedules, and reported outcomes. In response to peer review, we additionally performed an exploratory aggregate-data meta-analysis of the association between early postoperative ctDNA positivity and recurrence-related survival after curative-intent treatment of colorectal liver metastases. This quantitative analysis was added after peer review and was not prespecified in the original review protocol.

Studies were eligible for pooling if they reported an HR with a 95% confidence interval for postoperative ctDNA positivity and RFS or DFS, with a planned sampling time or a specified sampling window within 2–10 weeks after surgery. We included assays that selected mutations from tumor tissue or required plasma mutations to be confirmed in matched tumor tissue. Personalized multiplex assays, single-variant assays, and tissue-confirmed fixed panels were distinguished. Studies with sampling windows extending beyond this interval were excluded when results for the eligible time window could not be extracted separately. Only one early postoperative estimate per cohort was included. Estimates obtained after adjuvant chemotherapy, at the end of treatment, or during serial surveillance were not included in this analysis. Study-level HRs, sampling times, assay details, and extraction notes are provided in Supplementary Table S1. Eligibility decisions for the quantitative synthesis and reasons for excluding studies from pooling are provided in Supplementary Table S2.

Unadjusted and adjusted HRs were pooled separately. HRs were log-transformed, and standard errors were calculated from the reported 95% confidence intervals. We used an inverse-variance random-effects model, with restricted maximum likelihood estimation of between-study variance and modified Hartung–Knapp confidence intervals. The Hartung–Knapp variance factor was constrained to be at least 1, and confidence intervals were calculated using a t distribution with k−1 degrees of freedom, where k denotes the number of studies. Heterogeneity was assessed using Cochran’s Q, I^2^, and τ^2^. RFS and DFS were combined in the exploratory analysis, with a separate sensitivity analysis restricted to RFS.

We also examined a subgroup of studies using personalized 16-variant NGS assays. Sensitivity analyses were restricted to studies with pre-adjuvant chemotherapy sampling supported by the text or study protocol, with a further restriction to explicit statements in the text. We repeated the unadjusted analysis after omitting each study in turn and after substituting alternative HRs reported in the tables of Tie et al. [26] and Li et al. [27]. We also excluded Xu et al. [28] because the RFS definition and time origin were not clearly reported. In the adjusted analysis, Reinert et al. [29] and Xu et al. [28] were excluded separately and together to assess the effect of uncertainties in the reported comparison and endpoint definition, respectively.

We also repeated the unadjusted analysis using the DerSimonian–Laird estimator of between-study variance, retaining modified Hartung–Knapp confidence intervals.

## 3. Results

### 3.1. Study Characteristics

Twenty-eight studies published between 2018 and 2026 met the inclusion criteria and were included in the final synthesis [26,27,28,29,30,31,32,33,34,35,36,37,38,39,40,41,42,43,44,45,46,47,48,49,50,51,52,53]. These comprised prospective observational cohorts, retrospective single- and multicenter studies, translational sub-studies of randomized trials, including CAIRO5 and PRODIGE-14/METHEP-2, and data from the CIRCULATE-Japan GALAXY registry. The studies were conducted across Europe, Asia, North America, and Australia, with some involving multinational collaborations. Study populations varied considerably, including patients with upfront resectable liver-limited disease, initially unresectable disease treated with conversion therapy, and both synchronous and metachronous colorectal liver metastases. Perioperative treatment also differed across studies, ranging from surgery without preoperative chemotherapy to neoadjuvant, perioperative, or adjuvant systemic treatment [26,27,28,29,30,31,32,33,34,35,36,37,38,39,40,41,42,43,44,45,46,47,48,49,50,51,52,53].

After accounting for overlapping study populations, the studies represented 2079 unique patients. Overlap was identified among Wullaert et al. [38], Makrodimitris et al. [37], and Wullaert et al. [53] (MIRACLE cohort), Bolhuis et al. [32] and Crnovrsanin et al. [40] (CAIRO5), Reinert et al. [29] and Øgaard et al. [45], and Nishioka et al. [33] and Newhook et al. (2023) [34] (MD Anderson cohort). To avoid double counting, only the largest cohort from each overlapping population was used when calculating the total number of patients. In Raunkilde et al. [36], the 56 reported cases corresponded to 45 individual patients, as 11 patients were re-enrolled following recurrence. Sex was available for 1634 patients, of whom 1058 (64.7%) were men and 576 (35.3%) were women. Reported mean or median ages ranged from 52.2 to 69.0 years, while individual patient ages ranged from 18 to 92.5 years [26,27,28,29,30,31,32,33,34,35,36,37,38,39,40,41,42,43,44,45,46,47,48,49,50,51,52,53].

The main findings of the included studies are summarized in Table 1.

### 3.2. Risk of Bias

Of the 28 included studies, 17 (60.7%) were judged to have a moderate overall risk of bias and 11 (39.3%) a high overall risk of bias; none was classified as low risk. The main concerns involved incomplete adjustment for confounding, selective availability of blood samples, and limitations in statistical analysis, including data-driven cutoff selection and the handling of postoperative or longitudinal measurements. Outcome measurement was generally judged to be at low risk, although concerns arose in studies with less clearly defined recurrence criteria or intensified imaging following ctDNA detection. Domain-level assessments and study-specific justifications are presented in Supplementary Table S3.

### 3.3. ctDNA Detection and Sampling Strategies

Methodological execution varied significantly across the reviewed literature, with ctDNA detection strategies falling into four primary technological frameworks. Under the tumor-informed umbrella, platforms like Signatera or personalized SNV panels were built to track specific mutations found in a patient’s own sequenced tissue sample. On the other hand, tumor-naïve strategies bypassed this step entirely. They relied instead on predefined mutation kits or targeted droplet digital PCR (ddPCR) to hunt for common colorectal hotspots like KRAS, NRAS, and BRAF. Other teams shifted focus away from basic mutations; some evaluated epigenetic shifts by tracking methylation signatures—like SEPT9, NPY, or the TriMeth assay—while others looked at physical DNA structure using fragmentomics (DELFI-TF) or aneuploidy scores (mFast-SeqS). Outside of the raw technology, blood draw timing was equally non-standardized, spanning from a single, quick pre- or post-surgical check to tight long-term tracking throughout the entire therapy course [26,27,28,29,30,31,32,33,34,35,36,37,38,39,40,41,42,43,44,45,46,47,48,49,50,51,52,53].

Strategies for addressing hematopoietic variants differed across studies (Table 1). Reinert et al. [29] explicitly used matched white blood cell DNA to exclude variants arising from clonal hematopoiesis, whereas Wullaert et al. [38] performed selective testing and excluded three patients with GNAS variants. Newhook et al. [34] used a plasma-only variant classifier: specificity for recurrence was 100%, compared with 42.9% without filtering, and the classifier-based positive predictive value was 100%. These findings demonstrate the importance of variant attribution but do not establish that all unfiltered false-positive results arose from clonal hematopoiesis. In other studies, dedicated CHIP filtering was not explicitly described. The available reporting did not permit quantification of its independent effect on specificity or positive predictive value across platforms [29,34,38].

### 3.4. Preoperative ctDNA and Prognosis

The prognostic value of preoperative ctDNA varied across studies. Higher ctDNA levels were associated with shorter RFS and, in some studies, OS. Associations were more apparent in analyses using quantitative measures, such as methylation-derived tumor fractions, chromosomal aneuploidy scores, and plasma mutant allele frequencies. In the chemotherapy-naïve MIRACLE cohort, quantitative methylation and aneuploidy measures were associated with outcomes, whereas binary mutation-based ctDNA status was not significantly associated with prognosis. Other cohorts also reported no significant association between binary preoperative ctDNA status and recurrence or survival. Quantitative measures of preoperative ctDNA burden may provide additional prognostic information beyond a positive-or-negative classification, although differences in assays and prior systemic treatment limit comparisons between studies [26,27,28,29,30,31,32,33,34,35,36,37,38,39,40,41,42,43,44,45,46,47,48,49,50,51,52,53].

Interpretation of preoperative ctDNA also depends on whether blood was collected before or after systemic treatment. In Tie et al. [26], ctDNA detection and mutant allele fractions declined during neoadjuvant chemotherapy. A sample obtained after treatment therefore reflects a different clinical setting from an untreated baseline sample. Detection rates and prognostic associations should be considered separately for chemotherapy-naïve patients undergoing upfront resection, patients with resectable disease receiving neoadjuvant therapy, and initially unresectable patients receiving conversion therapy.

The clinical settings of the included cohorts differed substantially (Table 1). The MIRACLE studies and the CRLM subgroup of GALAXY evaluated patients undergoing upfront resection without preoperative chemotherapy for the current CRLM episode [38]. Tie et al. included patients with resectable disease managed with either upfront surgery or neoadjuvant chemotherapy, whereas Li et al. and Xu et al. included both initially resectable and conversion-treatment cohorts [26,27,28]. CAIRO5 and other conversion-treatment studies evaluated patients receiving systemic therapy before local treatment of initially unresectable disease. Several remaining studies reported preoperative chemotherapy without clearly distinguishing neoadjuvant from conversion intent [32]. These differences limit comparisons of preoperative ctDNA detection rates across studies [26,27,28,32,38].

Preoperative detection rates also varied considerably according to the assay used. Studies using tumor-informed or highly sensitive approaches generally reported high detection rates, often between 85% and 100%, whereas rates of approximately 60–65% were reported with some fixed, tumor-naïve mutation panels. This variation should be considered when comparing the prognostic value of preoperative ctDNA across studies, as differences in assay methodology and analytical sensitivity may contribute to the observed differences in detection rates [26,27,28,29,30,31,32,33,34,35,36,37,38,39,40,41,42,43,44,45,46,47,48,49,50,51,52,53].

### 3.5. Postoperative ctDNA and Recurrence Risk

Unlike preoperative findings, postoperative ctDNA showed a highly consistent link with patient outcomes across almost all literature. Finding detectable ctDNA after surgery consistently signals an elevated risk of relapse alongside poor RFS. The strength of this link fluctuated across cohorts, with hazard ratios spanning from a modest 2.0 to over 20.0, though values frequently clustered between 5.0 and 10.0. Furthermore, the positive predictive value for disease return hit 100% in multiple datasets. When looking at OS, post-surgical molecular positivity regularly mirrored shorter long-term survival trajectories. Crucially, this prognostic impact held firm across various multivariable models, remaining an independent risk factor even after accounting for node status, RAS/BRAF mutations, clinical risk scores, and the exact burden of liver metastases. Ultimately, data show that ctDNA outperforms classic serum markers like CEA in diagnostic accuracy [26,27,28,29,30,31,32,33,34,35,36,37,38,39,40,41,42,43,44,45,46,47,48,49,50,51,52,53].

The proportion of patients with detectable ctDNA after surgery ranged from approximately 20% to >60%, with differences in assay methodology and timing of blood collection across studies. Serial sampling also showed that ctDNA could become detectable before recurrence was identified on imaging, with reported lead times ranging from approximately 2.5 to >20 months [26,27,28,29,30,31,32,33,34,35,36,37,38,39,40,41,42,43,44,45,46,47,48,49,50,51,52,53]. One Danish cohort specifically evaluated ctDNA in patients with indeterminate findings on surveillance CT. Among these patients, ctDNA positivity was strongly associated with subsequent confirmation of recurrence, with all ctDNA-positive patients eventually developing recurrent disease. These findings suggest that ctDNA may provide additional information when surveillance imaging is inconclusive [29].

### 3.6. ctDNA Dynamics, Clearance, and Serial Monitoring

Several studies examined changes in ctDNA over time rather than relying on a single measurement. Across these studies, patients with persistently detectable ctDNA had the poorest outcomes, whereas those who remained ctDNA-negative throughout follow-up had the lowest risk of recurrence. Patients who became ctDNA-negative after surgery or systemic treatment generally had better outcomes than those with persistent ctDNA positivity, while conversion from a negative to a positive result during follow-up was associated with an increased risk of recurrence. Similar patterns were reported across studies using different ctDNA detection methods, including tumor-informed sequencing, methylation-based assays, and aneuploidy-based approaches [26,27,28,29,30,31,32,33,34,35,36,37,38,39,40,41,42,43,44,45,46,47,48,49,50,51,52,53].

A smaller number of studies also examined quantitative changes in ctDNA over time. Øgaard et al. evaluated ctDNA growth during postoperative surveillance and found that a higher ctDNA growth rate was associated with poorer OS [45]. Makrodimitris et al. assessed ctDNA burden before and after surgery using methylation- and copy-number-based tumor fraction estimates, alongside mutation-based measurements, allowing changes in ctDNA levels to be evaluated beyond a simple positive or negative result [37]. Together, these findings suggest that serial assessment of both ctDNA status and ctDNA levels may provide additional prognostic information compared with a single measurement [26,27,28,29,30,31,32,33,34,35,36,37,38,39,40,41,42,43,44,45,46,47,48,49,50,51,52,53].

### 3.7. ctDNA Status and Outcomes Following Adjuvant Chemotherapy

Several observational studies examined the association between postoperative ctDNA status and outcomes following adjuvant chemotherapy (ACT). In the CIRCULATE-Japan GALAXY analysis by Kataoka et al., treatment was not randomly assigned [41]. Among MRD-positive patients, ACT was associated with longer DFS than observation (adjusted HR 0.07, 95% CI 0.02–0.26; *p* < 0.0001). In MRD-negative patients, the association was not statistically significant (adjusted HR 0.68, 95% CI 0.29–1.58; *p* = 0.371). These subgroup findings do not establish that MRD status predicts treatment benefit, and the absence of statistical significance in MRD-negative patients should not be interpreted as evidence of no benefit. Xu et al. [28] also reported an association between ACT and longer RFS among patients with detectable ctDNA one month after liver resection. However, treatment allocation was non-randomized, which limits conclusions about whether ctDNA status predicts ACT benefit [28]. A CT was also associated with a higher rate of ctDNA clearance, and among ctDNA-positive patients who received ACT, clearance was associated with improved RFS (HR 0.214, 95% CI 0.065–0.70; *p* = 0.04). Earlier findings from Wang et al. were consistent with this pattern, showing that postoperative and post-ACT ctDNA status was associated with recurrence risk and that persistent ctDNA after ACT identified patients with particularly poor outcomes [48]. These findings are hypothesis-generating. Non-randomized treatment allocation, residual confounding, and potential time-related bias limit conclusions about ACT benefit. Landmark analyses, such as those used in GALAXY, address some timing-related concerns but do not remove treatment-selection bias. The predictive value of postoperative ctDNA and the clinical benefit of ctDNA-guided treatment therefore remain unproven in patients undergoing CRLM resection [26,27,28,29,30,31,32,33,34,35,36,37,38,39,40,41,42,43,44,45,46,47,48,49,50,51,52,53].

### 3.8. Assay Technology and Methodological Comparisons

Differences in assay methodology also influenced the prognostic information provided by ctDNA. Some studies assessed ctDNA quantitatively rather than simply classifying samples as positive or negative. Wang et al. [48], for example, found that higher baseline variant allele fraction (VAF) was associated with greater tumor burden, while a more than tenfold decrease in VAF during preoperative chemotherapy was associated with a better tumor response. Post-ACT ctDNA also showed better discrimination for RFS than the Clinical Risk Score (CRS), with an AUROC of 0.79 [26,27,28,29,30,31,32,33,34,35,36,37,38,39,40,41,42,43,44,45,46,47,48,49,50,51,52,53]. Similarly, Xu et al. reported higher AUROC values for ctDNA measured after ACT and one month after surgery than for CEA or pathological tumor regression grade [28].

The agreement between plasma ctDNA and matched tumor tissue varied between individual genes. In Wang et al. [48], concordance was 68% for TP53, 70% for APC, and 88% for KRAS, with higher rates reported for several less frequently altered genes. In the same study, tissue KRAS and BRAF mutation status was not significantly associated with RFS, whereas post-ACT ctDNA remained independently associated with outcome [48]. Overall, these findings show that the prognostic information provided by ctDNA depends not only on whether a mutation is present, but also on the amount of ctDNA detected and how it changes during treatment [26,27,28,29,30,31,32,33,34,35,36,37,38,39,40,41,42,43,44,45,46,47,48,49,50,51,52,53].

Approaches to excluding non-tumor-derived variants differed across mutation-based assays. Newhook et al. [34] used a predefined plasma variant classifier to distinguish tumor-derived from non-tumor-derived alterations; matched leukocyte sequencing was not reported. Within that cohort, specificity for recurrence was 42.9% with unfiltered plasma calls and 100% with the classifier, although this difference cannot be attributed specifically to CHIP removal. Wullaert et al. [38] tested white blood cell DNA in selected cases with similar preoperative and postoperative VAFs in genes associated with clonal hematopoiesis, leading to the exclusion of three patients with GNAS mutations. This was selective testing rather than systematic matched leukocyte sequencing in all patients. Jiang et al. [47] used a tumor-naïve Oncomine panel but did not report matched leukocyte sequencing or a dedicated CHIP-filtering procedure. The report by Nishioka et al. likewise did not describe matched leukocyte sequencing. These reporting differences limit direct comparisons of assay specificity [33,34,38,47].

### 3.9. Recurrence-Site Patterns

The clinical site of disease recurrence also seems to dictate how easily ctDNA can be picked up in plasma samples. Reinert et al. [29] successfully caught ctDNA in 86% of patients with liver recurrence, but that rate dropped to just 40% when the disease returned in the lungs. This means pulmonary relapses are much more likely to yield a false-negative blood test. The CIRCULATE-Japan GALAXY cohort reported a similar pattern; liver metastases were highly prevalent in the MRD-positive group, whereas 61.9% of the failures in the MRD-negative group occurred exclusively in the lungs [41]. Jiang et al. also documented higher post-surgical ctDNA positivity prior to liver recurrence compared to other anatomical locations like the peritoneum or lungs [47].

The lower detection rate in pulmonary recurrence may be related to the small volume of disease at the time of radiological detection. In the Reinert cohort, the mean diameter of lung metastases was only 8.1 mm, and the authors considered low ctDNA shedding from these small lesions the most likely explanation for the negative plasma results [29]. Li et al. [27] reported a similar observation, noting that small lung lesions may be visible on imaging before they release sufficient ctDNA to be detected in plasma [27]. These findings are important when interpreting a negative ctDNA result during surveillance, particularly in patients who later develop small-volume pulmonary recurrence [26,27,28,29,30,31,32,33,34,35,36,37,38,39,40,41,42,43,44,45,46,47,48,49,50,51,52,53].

### 3.10. Exploratory Meta-Analysis

**Figure 2:**
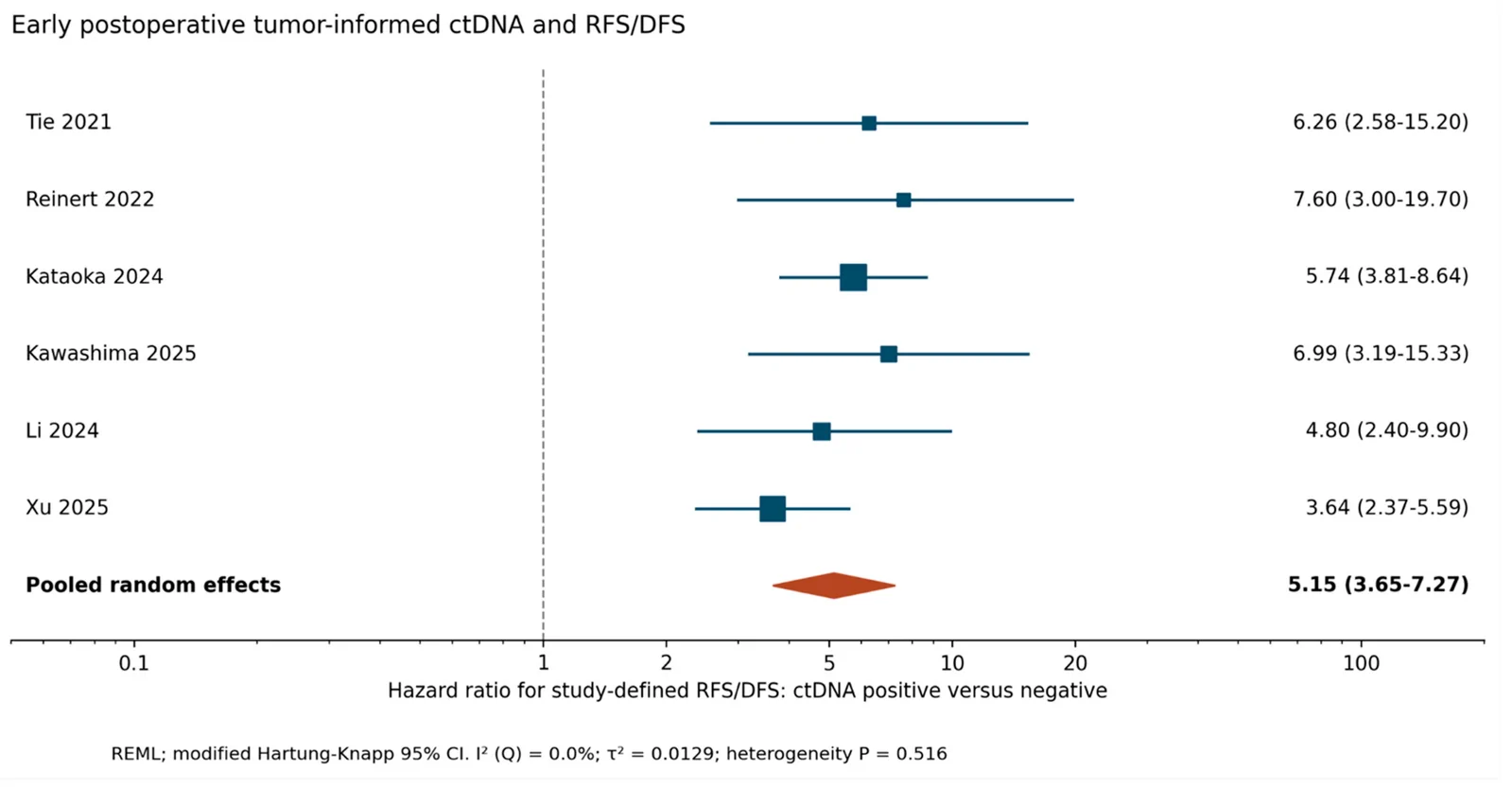
Association between early postoperative ctDNA positivity and study-defined RFS/DFS in six studies using tissue-informed assays. Unadjusted HRs compare ctDNA-positive with ctDNA-negative patients [26,27,28,29,41,42]. Square sizes represent study weights, horizontal lines indicate 95% confidence intervals, and the diamond represents the pooled estimate. Pooling used a random-effects model with REML estimation and modified Hartung–Knapp confidence intervals. Assays and endpoint definitions differed between studies.

Six studies provided unadjusted HRs for the association between early postoperative ctDNA positivity and recurrence-related survival [26,27,28,29,41,42]. Five reported RFS and one reported DFS. Postoperative ctDNA positivity was associated with poorer outcomes, with a pooled HR of 5.15 (95% CI 3.65–7.27) (Figure 2). Statistical heterogeneity was not detected by Cochran’s Q (Q = 4.24, df = 5, *p* = 0.516; I^2^ = 0%), while the REML estimate of τ^2^ was 0.0129. The studies nevertheless differed in assay technology, patient populations, and endpoint definitions.

Two studies used personalized 16-variant NGS assays, although the platforms differed [27,41]. Pooling these studies yielded an HR of 5.49 (95% CI 0.55–54.64) (Figure S1). The wide confidence interval reflects the uncertainty of pooling only two studies and does not allow a precise estimate for this assay subgroup.

Five studies provided adjusted HRs, which were pooled separately from the unadjusted estimates [26,27,28,29,41]. The pooled adjusted HR was 4.95 (95% CI 2.45–10.01) (Figure S2), with heterogeneity across studies (I^2^ = 60.5%; τ^2^ = 0.1714). The adjustment variables differed between studies, limiting the comparability of these estimates.

In sensitivity analyses of the unadjusted estimates, restricting pooling to RFS yielded an HR of 4.98 (95% CI 3.08–8.05). Restriction to studies with pre-adjuvant chemotherapy sampling supported by the text or study protocol yielded an HR of 4.97 (95% CI 3.35–7.39) (Figure S3). Excluding Xu et al. [28] yielded an HR of 5.93 (95% CI 3.94–8.93). In the adjusted analysis, excluding Reinert et al. [29], Xu et al. [28], or both yielded HRs of 4.41 (95% CI 1.90–10.20), 6.46 (95% CI 3.54–11.78), and 5.99 (95% CI 2.43–14.75), respectively.

Restricting the unadjusted analysis to the three studies that explicitly described sampling before adjuvant chemotherapy yielded an HR of 6.04 (95% CI 2.83–12.90). Using the alternative table estimates reported by Tie et al. [26] and Li et al. [27] yielded an HR of 5.17 (95% CI 3.66–7.30). In leave-one-out analyses, pooled unadjusted HRs ranged from 4.97 to 5.93, with all corresponding confidence intervals remaining above 1. Pooled estimates and heterogeneity statistics for all analyses are provided in Supplementary Table S4.

The certainty of evidence that early postoperative ctDNA positivity is associated with shorter RFS/DFS was rated as moderate for both the unadjusted and adjusted analyses, with downgrading for risk of bias (Supplementary Table S5). Although adjusted estimates varied in magnitude, their direction remained consistent, including in exclusion analyses. These ratings concern the presence of a prognostic association and do not establish a precise effect size or a benefit from ctDNA-guided treatment or surveillance.

## 4. Discussion

Postoperative ctDNA was consistently associated with recurrence after CRLM resection, despite differences in patient populations, treatments, and assays. Serial testing provided further prognostic information, as persistent or newly detected ctDNA during follow-up was associated with recurrence and often preceded radiological detection. In contrast, the prognostic value of preoperative ctDNA was less consistent. Observational studies have also examined outcomes following adjuvant chemotherapy according to postoperative MRD status. These findings are hypothesis-generating and do not establish that ctDNA predicts treatment benefit [26,27,28,29,30,31,32,33,34,35,36,37,38,39,40,41,42,43,44,45,46,47,48,49,50,51,52,53].

Our findings are broadly consistent with those of Vail et al. [54], who previously examined the prognostic role of postoperative cfDNA in 10 studies including 669 patients with resected CRLM. They reported that positive postoperative cfDNA carried a more than threefold higher risk of relapse (pooled HR 3.11, 95% CI 2.29–4.22). By building on a much larger and more recent pool of evidence, our review validates this link while adding critical new data on serial ctDNA trends, assay differences, and how the site of recurrence affects testing. We found that persistent ctDNA or its return during follow-up points to a very high risk of recurrence, while ctDNA clearance signals a much better outlook. The earlier review focused mainly on single postoperative timepoints, leaving these long-term tracking patterns largely unexamined. Still, it is important to note that a negative ctDNA result does not completely rule out a return of the disease. Vail et al. [54] observed that roughly one-third of cfDNA-negative patients still recurred within the first year, a rate that climbed over time. Our findings help explain this gap by showing that assay sensitivity drops when dealing with small-volume disease, particularly small lung recurrences [26,27,28,29,30,31,32,33,34,35,36,37,38,39,40,41,42,43,44,45,46,47,48,49,50,51,52,53,54].

The more recent literature also begins to address a different question: whether ctDNA can help identify patients who are more likely to benefit from postoperative chemotherapy. This is particularly relevant in CRLM, where the benefit of perioperative chemotherapy remains difficult to define. Bosma et al. [55] found a significant improvement in DFS with perioperative chemotherapy, but no corresponding improvement in OS. Against this background, ctDNA may offer a way to move beyond treating patients solely on the basis of conventional clinicopathological risk. Vail et al. [54] had already proposed that cfDNA-positive patients might represent a group in whom treatment intensification should be explored. Some included studies reported an association between ACT and better outcomes among patients with postoperative ctDNA positivity. However, differences in statistical significance between ctDNA-positive and ctDNA-negative subgroups do not establish a difference in treatment effect. Non-randomized treatment allocation, residual confounding, and potential time-related bias limit interpretation of these findings. These observations are clinically interesting, but the evidence is still limited to non-randomized analyses. For now, ctDNA appears better established as a marker of recurrence risk than as a tool for selecting postoperative treatment, and prospective randomized trials will be needed to determine whether ctDNA-guided therapy can improve outcomes [26,27,28,29,30,31,32,33,34,35,36,37,38,39,40,41,42,43,44,45,46,47,48,49,50,51,52,53,55].

The less consistent findings for preoperative ctDNA may partly reflect differences in treatment exposure and in the way ctDNA was measured. Recent studies in chemotherapy-naïve patients suggest that the amount of ctDNA present before surgery may carry prognostic information. In the MIRACLE cohort, higher presurgical ctDNA levels were associated with shorter RFS and OS and with a greater risk of early multiorgan recurrence [38]. Similarly, Makrodimitris et al. found that methylation-based estimates of preoperative ctDNA burden were independently associated with both RFS and OS [37]. In contrast, Moretto et al., who studied patients undergoing resection after first-line systemic treatment, found that presurgical ctDNA status alone was not significantly associated with RFS, whereas a reduction in ctDNA from baseline to surgery was associated with better outcomes [39]. These differences suggest that the meaning of a preoperative ctDNA result may depend on when it is measured in relation to systemic treatment. In untreated patients, the absolute ctDNA level may provide information on disease burden and biology, whereas after systemic therapy, the change in ctDNA over time may be more informative than a single presurgical measurement [37,38,39].

Beyond its prognostic value, an important question is how ctDNA could fit alongside the tools already used in clinical practice. Rather than replacing conventional clinical, pathological, or radiological assessment, ctDNA may be more useful as an additional source of information. Bolhuis et al. found a close association between postoperative ctDNA and pathological response, while other studies showed that ctDNA remained prognostic even after established clinicopathological factors were taken into account [32]. Nishioka et al. also found that multiple liver metastases and RAS/TP53 co-mutation were independently associated with postoperative ctDNA positivity, suggesting that ctDNA detection may be influenced by both disease burden and the underlying biology of the tumor [33]. Unlike most conventional risk factors, however, ctDNA can be measured repeatedly and may therefore provide a more dynamic picture of residual disease as patients move through treatment and follow-up [32,33].

There are also practical issues that need to be considered when interpreting postoperative ctDNA. One of these is the timing of blood collection. Surgery can temporarily increase the amount of cfDNA in the circulation, which may dilute the tumor-derived fraction and make ctDNA more difficult to detect in the early postoperative period. Bolhuis et al. [32] noted that this effect may persist for several weeks and discussed repeat sampling after four weeks in patients with an initially negative postoperative result. This may partly explain the wide range of postoperative ctDNA detection rates across the included studies. The site of recurrence also appears to matter, particularly for small-volume pulmonary disease, where ctDNA was less frequently detected. A negative result should therefore not be viewed as evidence that residual disease is absent, especially when it is based on a single early postoperative sample. This is also one of the reasons why serial monitoring may ultimately be more informative than testing at a single predefined timepoint [26,27,28,29,30,31,32,33,34,35,36,37,38,39,40,41,42,43,44,45,46,47,48,49,50,51,52,53].

Organ-specific differences in ctDNA shedding may also contribute to the lower detection rates observed in pulmonary recurrence. Beyond lesion size, the release of tumor DNA into the circulation may depend on tumor vascularization, vascular invasion, cell turnover and necrosis. Studies in metastatic CRC have reported lower plasma ctDNA fractions in lung-only than in liver-only disease, while pathological studies in primary lung cancer provide indirect evidence linking shedding to vascular invasion and necrosis. However, the included CRLM studies did not establish the relative contribution of these mechanisms, and a specific role for the blood–air barrier remains unproven. Clinically, a negative ctDNA result should not delay imaging when pulmonary recurrence is suspected. Chest CT and, where appropriate, interval imaging or tissue confirmation should be guided by the radiological findings and clinical context. Repeat ctDNA testing may provide additional information but should not replace imaging-based surveillance [29,56].

The exploratory meta-analysis supports an association between postoperative ctDNA positivity and poorer recurrence-related outcomes. However, the absence of detected statistical heterogeneity in the unadjusted analysis does not remove the clinical and methodological differences between studies. The adjusted analysis showed greater heterogeneity, while the subgroup of personalized multiplex NGS assays included only two studies and yielded a highly imprecise estimate. Given the moderate-to-high risk of bias across the included studies, these findings support the prognostic value of ctDNA but do not establish a predictive benefit from adjuvant chemotherapy or show that ctDNA-guided management improves patient outcomes.

Clonal hematopoiesis can produce false-positive results in mutation-based plasma assays because variants such as TP53 and, less frequently, KRAS may originate from hematopoietic cells rather than the tumor. Matched leukocyte analysis can help establish their origin, whereas computational filtering and tumor-tissue matching provide different safeguards and should not be treated as equivalent procedures. Inadequate filtering may reduce specificity and positive predictive value, but the included studies do not allow the magnitude of this effect to be quantified. Positive predictive value also depends on recurrence frequency and follow-up duration. Consequently, differences in reported performance cannot be attributed to assay platform alone, and the absence of reported leukocyte testing should be treated as a methodological uncertainty rather than proof of CHIP-related false positives [57].

This review has several limitations. Most included studies were observational and relatively small, with considerable differences in patient populations, ctDNA assays, sampling timepoints, and treatment strategies. These differences limited direct comparisons between studies. All included studies were judged to have a moderate or high overall risk of bias, limiting confidence in the magnitude of the reported prognostic associations. Incomplete adjustment for confounding, selective sample availability, and limitations in statistical analysis may have influenced these estimates. The association between postoperative ctDNA positivity and recurrence therefore does not establish that ctDNA-guided treatment or surveillance improves clinical outcomes. Although selected studies could be pooled in an exploratory meta-analysis, differences in assays, patient populations, and outcome definitions limit the interpretation of the pooled estimates. Some reporting uncertainties also remained. In Xu et al. [28], the exact definition and time origin of RFS were not clearly stated. In Reinert et al. [29], the direction of the adjusted HR was unclear from the comparison label in the study’s supplementary table. We therefore interpreted it using the main text and survival figure; sensitivity analyses excluding this estimate were also performed. Exclusion analyses were therefore performed, but these do not resolve the underlying reporting uncertainties. In addition, sampling diagrams indicated planned rather than verified patient-level timing before adjuvant chemotherapy. Follow-up was also relatively short in some of the more recent studies, particularly for OS. Finally, differences in ctDNA shedding, especially in patients with small pulmonary recurrences, may affect the interpretation of negative results.

Future research should focus on how ctDNA can be used in everyday clinical practice after CRLM resection. Prospective studies with more consistent assays and sampling schedules are needed, particularly to determine whether serial ctDNA monitoring can help guide adjuvant treatment and follow-up. Most importantly, randomized trials are needed to show whether changing treatment according to ctDNA results can ultimately improve patient outcomes. The PRISMA 2020 checklist is provided in the Supplementary Materials Table S6.

## 5. Conclusions

Postoperative ctDNA positivity is associated with a higher risk of recurrence after CRLM resection, and serial measurements may provide additional prognostic information. The exploratory meta-analysis supports this association, but differences in assays, sampling schedules, patient populations, and outcome definitions limit its interpretation. All included studies had a moderate or high overall risk of bias. Current evidence does not establish that ctDNA predicts benefit from adjuvant chemotherapy or that using it to guide treatment or surveillance improves outcomes. Randomized trials with clearly defined sampling and treatment protocols are needed before ctDNA-guided management can be recommended for routine practice.

## Figures and Tables

**Table 1:** Main characteristics and key findings of the included studies.

Study	Country/Design	Population	ctDNA Assay	Sampling	Key Findings
Benešová et al. 2019 [30]	Czech Republic; prospective multicenter study	*n* = 47; liver-only 35, liver ± other sites 12; M/F 31/16; mean 63.6 ± 12.3 y Preoperative systemic treatment and initial resectability were insufficiently reported to distinguish groups A, B, and C.	DCE mutation panel (KRAS, TP53, APC, PIK3CA, BRAF, CTNNB1)	Pre-op; ≤1 week post-op; ~q3-month follow-up	Postoperative ctDNA reflected resection radicality. In 22 documented recurrences after R0 liver-metastasis resection, ctDNA was detected in 22/22 (100%), compared with 16/22 (73%) by imaging and 15/22 (68%) by tumor markers.
He et al. 2020 [31]	China; prospective single-center study	*n* = 20; M/F 14/6; mean 55 ± 13 y Mixed preoperative-treatment cohort: 11/20 patients received preoperative chemotherapy, described as neoadjuvant therapy. Initial resectability and conversion intent were not separately reported.	Capture-based targeted deep NGS (41-gene ColonCore panel)	Within 7 days pre-op and within 7 days post-op	Preoperative ctDNA was detected in 17/20 (85%), correlated with tumor burden (R = 0.679), and was associated with shorter PFS. Only 4/20 remained ctDNA-positive after surgery, and postoperative status was not significantly associated with PFS.
Bolhuis et al. 2021 [32]	Netherlands; prospective translational CAIRO5 substudy	*n* = 23; M/F 15/8; median 63 y Group C: initially unresectable CRLM treated with conversion systemic therapy before resection.	KRAS/NRAS hotspot ddPCR	Baseline; pre-op; post-op; q3-month follow-up	Postoperative ctDNA was detected in 6/23 (26%) and was associated with shorter RFS (HR 3.3; adjusted HR 4.1). Detectable ctDNA was also strongly associated with poor pathological response.
Nishioka et al. 2022 [33]	USA; retrospective single-center cohort	*n* = 105; M/F 63/42; median 54 y Mixed preoperative-treatment cohort: 86% received preoperative chemotherapy. Initial resectability and conversion intent were not separately reported.	Plasma NGS (Guardant 23- or 70-gene panel)	Post-op within 180 days	Postoperative ctDNA was positive in 32/105 (30%) and was associated with shorter RFS and OS. It was the only independent predictor of recurrence within 1 year (HR 11.8); multiple CRLM and RAS/TP53 co-mutation independently predicted ctDNA positivity.
Newhook et al. 2023 [34]	USA; prospective single-center cohort	*n* = 48; sex/age NR in main text All patients received preoperative chemotherapy before curative-intent hepatectomy. Initial resectability and conversion intent were not separately reported	Plasma NGS with tumor-informed and tumor-uninformed classifiers A proprietary plasma-only variant classifier was used to distinguish tumor-derived from non-tumor-derived alterations; matched leukocyte sequencing was not reported. Tumor-tissue filtering was evaluated separately in 38 patients.	Paired pre-op and post-op samples	Postoperative, but not preoperative, ctDNA was associated with shorter RFS and OS. Persistent positivity had the poorest outcomes, whereas perioperative ctDNA clearance was associated with a substantially lower recurrence risk.
Chen, Ji, Xu et al. 2024 [35]	China; retrospective observational cohort	181 enrolled; 111 resected with POST samples; M/F 84/27 in POST cohort; median ~55 y Group C: initially unresectable CRLM treated with conversion systemic therapy before curative-intent local treatment.	Tumor-agnostic methylation ddPCR (SEPT9/NPY) Corresponding leukocyte samples were used to assess background SEPT9/NPY methylation during marker validation; mutation-based CHIP filtering was not applicable to this assay.	Baseline; pre-op; post-op; EOT; progression	POST and EOT methylated ctDNA positivity were strongly associated with shorter RFS (HR 9.44 and 11.48, respectively) and remained independent predictors on multivariable analysis. Baseline and preoperative status were not significantly prognostic.
Raunkilde et al. 2025 [36]	Denmark; prospective observational study	56 treatment episodes from 45 unique patients; case-level M/F 17/39; median 67 y Mixed preoperative-treatment cohort: neoadjuvant systemic therapy was reported in 37/56 treatment episodes (66%). Initial resectability and conversion intent were not separately reported	Multiplex methylation ddPCR (NPY, KANK1, GAL3ST3) Methylation-based ddPCR targeting NPY, KANK1, and GAL3ST3; mutation-based CHIP filtering was not applicable.	Pre-op and post-op In patients receiving neoadjuvant systemic therapy, the preoperative sample was collected after completion of treatment.	Both preoperative and postoperative meth-ctDNA were associated with PFS. Postoperative meth-ctDNA positivity was also associated with poorer OS (HR 4.1).
Makrodimitris et al. 2026 [37]	Netherlands; prospective MIRACLE cohort analysis	154 included; pre-op profiles *n* = 120, post-op *n* = 117; M/F 81/39 in pre-op cohort; median ~66 y Group A: chemotherapy-naïve, resectable CRLM treated upfront; MIRACLE cohort.	Genome-wide cfDNA methylation (MeD-seq) plus CNV profiling ctDNA fractions were estimated from genome-wide cfDNA methylation and copy-number profiles using healthy-control and CRLM-tissue references; mutation-based CHIP filtering was not applicable to these estimates.	<24 h pre-op; 3 weeks post-op	Preoperative methylation-derived tumor fraction independently predicted RFS (HR 1.20 per 0.1 increase) and OS (HR 1.31). Higher postoperative methylation-derived ctDNA burden was associated with shorter RFS.
Wullaert et al. 2026 [38]	Netherlands; MIRACLE cohort analysis	*n* = 182; M/F 126/56; median 66.5 y Group A: resectable CRLM treated upfront, without neoadjuvant or adjuvant chemotherapy for the CRLM episode.	mFast-SeqS aneuploidy score Genome-wide aneuploidy was assessed using mFast-SeqS relative to healthy controls; mutation-based CHIP filtering was not applicable to this assay.	Single presurgical sample	High presurgical aneuploidy score was associated with early multiorgan recurrence (44% vs. 13%) and remained independently associated with multiorgan recurrence (HR 2.56), as well as poorer RFS and OS.
Moretto et al. 2026 [39]	Italy; retrospective multicenter cohort	*n* = 116 presurgery-evaluable; M/F 64/52; age reported by ctDNA subgroup Group C: initially unresectable, liver-limited metastatic CRC resected after first-line conversion systemic therapy.	Tumor-naïve Tempus xM MRD assay Tempus xM combined methylation and genomic variant analysis. ctDNA status and PTFM estimates were methylation-based, whereas exploratory VAF analyses used genomic variants. Matched leukocyte sequencing for CHIP filtering was not reported.	Baseline; presurgery; postsurgery	Presurgery ctDNA status was not associated with RFS, whereas a ≥50% VAF reduction from baseline to surgery predicted longer RFS. Postsurgery ctDNA positivity strongly predicted recurrence (HR 6.66; adjusted HR 4.15).
Crnovrsanin et al. 2026 [40]	Netherlands/USA/Germany; translational CAIRO5 analysis	*n* = 101 overall; *n* = 46 achieved complete local treatment and entered early-recurrence analysis; M/F 62/39; median 59 y Group C setting: initially unresectable, liver-limited disease treated with induction systemic therapy; resection outcomes assessed in the locally treated subset.	Mutation-independent fragmentomics (DELFI-TF) DELFI-TF estimated ctDNA from cfDNA fragmentation profiles independently of tumor tissue and mutation detection; mutation-based CHIP filtering was not applicable to this assay.	Baseline and on-treatment	Baseline DELFI-TF and its on-treatment change were independently associated with OS in the full cohort. In the 46 patients completing local treatment, baseline and on-treatment DELFI-TF also predicted early recurrence.
Kataoka et al. 2024 [41]	Japan/USA; CIRCULATE-Japan GALAXY analysis	*n* = 190; M/F 118/72; median 68 y Group A: upfront resection without preoperative chemotherapy for the current CRLM episode.	Tumor-informed 16-plex mPCR-NGS (Signatera) Whole-exome sequencing of tumor tissue and matched normal DNA from peripheral blood was used to select up to 16 tumor-specific somatic variants; ctDNA positivity required detection of at least two variants.	Presurgery; MRD window (2–10 weeks); serial surveillance	MRD positivity was a strong independent predictor of DFS (adjusted HR 7.32) and was associated with poorer OS. ACT improved DFS in MRD-positive patients (adjusted HR 0.07) but not in MRD-negative patients (adjusted HR 0.68).
Kawashima et al. 2025 [42]	Japan; single-institution observational study	*n* = 53; M/F 30/23; age reported by category Preoperative systemic treatment and initial resectability were insufficiently reported to distinguish groups A, B, and C.	Tumor-informed targeted NGS followed by patient-specific ddPCR Tumor-tissue sequencing was used to select mutations for plasma ddPCR tracking; matched leukocyte testing for CHIP filtering was not reported.	Immediately pre-op and 21–30 days post-op	All 11 MRD-positive patients recurred, including 9/11 with early recurrence. MRD positivity was associated with shorter RFS (HR 6.99) and OS (HR 5.19); preoperative ctDNA burden showed a weaker association with outcome.
Montalti et al. 2025 [43]	Italy; prospective cohort	*n* = 51; M/F 31/20; mean 62.4 ± 11 y Mixed preoperative-treatment cohort: 24/51 patients (47.1%) received preoperative chemotherapy. Initial resectability and conversion intent were not separately reported.	Tumor-agnostic targeted NGS liquid-biopsy panel Matched tumor-tissue NGS was used to support variant attribution in 15 patients; matched leukocyte sequencing for CHIP filtering was not reported	24 h pre-op; 48 h post-op; 30 days post-op	Preoperative molecular-signature status was not significantly associated with DFS or OS. Postoperative persistence independently predicted recurrence (HR 2.77) and was associated with shorter DFS, but not with OS.
Narayan et al. 2019 [44]	USA; prospective single-center pilot cohort	*n* = 59; M/F 41/18; median 52.2 y Resectable CRLM at surgery; preoperative systemic treatment and initial resectability were insufficiently reported to distinguish groups A, B, and C.	Targeted hybrid-capture NGS (9-gene plasma panel) Matched blood DNA was used to exclude germline variants; a dedicated strategy for filtering clonal hematopoiesis–derived variants was not explicitly reported	Intra-op peripheral, portal and hepatic veins; peripheral sample 3–4 weeks post-op	Peripheral blood closely reflected portal and hepatic-vein ctDNA profiles, while tissue–plasma agreement was lower. Pre-resection peripheral TP53-mutant ctDNA was associated with worse 2-year disease-specific survival (79% vs. 90%, *p* = 0.024).
Reinert et al. 2022 [29]	Denmark; prospective single-center cohort	*n* = 68 evaluable; M/F 41/27; median 67.7 y Mixed preoperative-treatment cohort: 54 patients received no neoadjuvant chemotherapy for CRLM and 14 received neoadjuvant chemotherapy. Initial resectability and conversion intent were not separately reported.	Tumor-informed patient-specific ddPCR Matched white blood cell DNA was used for each patient to exclude variants arising from clonal hematopoiesis.	Pre-op; day-30 post-op; q3-month surveillance	Day-30 ctDNA positivity predicted recurrence (HR 7.6). Serial ctDNA preceded imaging by a median of 2.5 months; at indeterminate CT findings, ctDNA positivity had a PPV of 100%, while lung recurrences were more often ctDNA-negative.
Øgaard et al. 2022 [45]	Denmark; prospective single-center cohort	*n* = 96; M/F 64/32; median 67 y Mixed preoperative-treatment cohort: 9/96 patients received neoadjuvant chemotherapy and 3/96 received perioperative chemotherapy. Initial resectability and conversion intent were not separately reported.	Tumor-agnostic TriMeth methylation ddPCR TriMeth used methylation-based multiplex ddPCR targeting C9orf50, CLIP4, and KCNQ5; mutation-based CHIP filtering was not applicable to this assay.	Pre-op; post-op; post-ACT/EOT; serial surveillance	Preoperative ctDNA was not associated with recurrence. Postoperative and post-ACT positivity predicted shorter RFS (HR 4.5 and 8.4); serial testing led imaging by a median 3.1 months and had PPV/NPV 100%/75% at inconclusive CT.
Iwai et al. 2020 [46]	Japan; prospective single-center cohort	*n* = 41; 60.9% male; median 69 y Preoperative systemic treatment and initial resectability were insufficiently reported to distinguish groups A, B, and C.	cfDNA integrity marker (LINE-1/β-globin ratio; cLBR)—not a tumor-specific ctDNA assay The assay measured the long cfDNA fragment/β-globin ratio by qPCR rather than tumor-specific mutations; mutation-based CHIP filtering was not applicable.	Pre-hepatectomy and 1 month post-op	An increase in cLBR after hepatectomy was associated with early recurrence and independently predicted recurrence (HR 3.90). Preoperative cfDNA measures were not prognostic.
Jiang et al. 2023 [47]	China; prospective cohort	*n* = 67; M/F 38/29; age reported by category Resectable CRLM at enrollment; prior systemic treatment and initial resectability were insufficiently reported to distinguish groups A, B, and C	Tumor-naïve Oncomine Colon cfDNA NGS Tumor-naïve mutation-based testing; matched leukocyte sequencing or a dedicated CHIP-filtering strategy was not reported.	Single postoperative sample 3–6 weeks after resection	Postoperative ctDNA was positive in 15/67 (22.4%) and was associated with shorter RFS (HR 3.60) and early recurrence. Higher mutant allele fraction further stratified risk; no ctDNA was detected in the eight lung-only recurrences.
Tie et al. 2021 [26]	Australia; prospective multicenter cohort	*n* = 54; M/F 38/16; median 64 y Mixed A/B cohort: resectable CRLM; upfront resection (*n* = 31) or neoadjuvant chemotherapy followed by resection (*n* = 23).	Tumor-informed personalized mutation tracking Leukocyte DNA was used to exclude constitutional polymorphisms; a dedicated CHIP-filtering strategy was not explicitly reported.	Baseline; on-treatment; post-op; EOT; serial	Baseline ctDNA was not significantly associated with RFS or OS. Postoperative positivity predicted poorer 5-year RFS (HR 6.26) and OS (HR 4.2), while EOT positivity identified an especially high-risk group.
Wang et al. 2021 [48]	China; prospective single-center cohort	*n* = 91; sex/age not reported in available main-text extract Mixed preoperative-treatment cohort: 84/91 patients received preoperative chemotherapy and 7/91 underwent surgery without it. Initial resectability and conversion intent were not separately reported.	Tumor-informed broad-panel sequencing with tissue–plasma comparison Peripheral blood lymphocyte DNA was extracted, and a healthy-donor panel of normals was used for variant calling; a dedicated CHIP-filtering strategy was not explicitly reported.	Baseline; pre-op; post-op; post-ACT; progression	A ≥10-fold fall in VAF during preoperative chemotherapy was associated with better radiological response. Postoperative and post-ACT ctDNA positivity were associated with higher recurrence rates and shorter RFS; post-ACT status had an AUROC of 0.79.
Bhangu et al. 2018 [49]	Austria; prospective non-randomized single-center study	*n* = 34 receiving neoadjuvant/conversion chemotherapy; M/F 24/10; median 64 y Mixed B/C cohort: all 34 patients received preoperative systemic chemotherapy, including patients with initially resectable disease and patients undergoing conversion therapy; 22 subsequently underwent liver resection.	Tumor-agnostic methylation assays (SEPT9 plus AIT marker panel) Methylation-based testing of SEPT9 and a 47-gene methylation panel; mutation-based CHIP filtering was not applicable to these assays.	Baseline and before each chemotherapy cycle	Methylation markers correlated more closely with tumor volume than CEA/CA19-9 and distinguished patients who ultimately underwent surgery from those who did not. Early decreases were associated with pathological response; postoperative recurrence/survival were not assessed.
Bidard et al. 2019 [50]	France; prospective PRODIGE-14/METHEP-2 ancillary study	*n* = 153 overall; baseline ctDNA *n* = 125; R0/R1 liver resection *n* = 91; median 60 y Conversion-treatment setting: potentially resectable liver metastases treated with first-line systemic therapy to enable resection.	KRAS exon-2 ddPCR (with parallel CTC analysis) Plasma KRAS mutations were assessed by droplet digital PCR, with tumor-tissue KRAS status available; matched leukocyte testing for CHIP filtering was not reported.	Baseline; 4 weeks on treatment; presurgery	Baseline KRAS ctDNA showed 91% sensitivity versus tissue. Lower ctDNA at 4 weeks was associated with eventual R0/R1 resection; among resected patients, detectable presurgical ctDNA was associated with markedly shorter OS.
Polivka et al. 2020 [51]	Czech Republic; retrospective single-center cohort	*n* = 71 overall; pre-op plasma *n* = 30; paired pre/post plasma *n* = 17; M/F 42/29; mean 62.7 y Preoperative systemic treatment and initial resectability were insufficiently reported to distinguish groups A, B, and C.	KRAS G12/G13 ddPCR KRAS G12/G13 mutations were assessed by ddPCR in plasma and tumor tissue; matched leukocyte testing or a dedicated CHIP-filtering strategy was not reported.	Pre-op and early post-op	Tissue KRAS status was not prognostic, whereas preoperative plasma KRAS fractional abundance independently predicted DFS and OS. All 17 patients with paired pre/postoperative plasma became KRAS-negative after liver surgery.
Kalil et al. 2024 [52]	Canada; retrospective single-center cohort	*n* = 29 analyzed; M/F 17/12; median 61 y Mixed preoperative-treatment cohort: 24/29 patients (83%) received preoperative chemotherapy, described as neoadjuvant therapy. Initial resectability and conversion intent were not separately reported.	KRAS/PIK3CA digital PCR Plasma digital PCR targeted KRAS and/or PIK3CA mutations identified by tumor-tissue NGS; matched leukocyte testing for CHIP filtering was not reported.	Pre-op; 1–3 months post-op; q3–6-month surveillance	All 8 postoperative ctDNA-positive patients recurred. Postoperative positivity was associated with shorter DFS (HR 2.95) and markedly poorer OS (HR 23.7); preoperative ctDNA was less clearly associated with recurrence.
Li Y, Xu J et al. 2024 [27]	China; prospective single-center cohort	*n* = 60 eligible; 70% male; median 59 y Mixed A/B/C cohort: initially resectable disease (*n* = 33; 6 received neoadjuvant chemotherapy) and initially unresectable disease treated with conversion chemotherapy (*n* = 27).	Tumor-informed personalized 16-SNV MRD assay A personalized 16-SNV panel was designed using tumor-tissue WES and matched white blood cell DNA as the normal control; positivity required at least two detected variants. A dedicated CHIP-filtering procedure was not explicitly described.	Pre-op; day-30 post-op; post-ACT/EOT; q3-month surveillance	Postoperative ctDNA was the only independent predictor of RFS (HR 5.1). Longitudinal testing detected recurrence a median of 3.5 months before imaging, and ctDNA clearance after ACT was associated with longer RFS.
Wullaert et al. 2025 [53]	Netherlands; prospective multicenter MIRACLE cohort	*n* = 188; M/F 130/58; median 67 y Group A: chemotherapy-naïve, resectable CRLM treated upfront; MIRACLE cohort.	Oncomine Colon cfDNA NGS at baseline; postoperative dPCR Selective white blood cell DNA testing was performed in cases with similar pre- and postoperative VAFs in genes associated with clonal hematopoiesis; three patients with GNAS variants were excluded.	Pre-op and post-op (median 18 days)	Baseline ctDNA was not significantly associated with 1-year RFS. Postoperative ctDNA independently predicted shorter RFS (HR 2.35) after adjustment for the Fong risk score and primary-tumor location.
Xu D et al. 2025 [28]	China; prospective single-center cohort	*n* = 297 analyzed overall; 1-month ctDNA subset *n* = 141 (M/F 100/41) Mixed A/B/C cohort. Among patients with one-month ctDNA results (*n* = 141): no preoperative chemotherapy (*n* = 36), neoadjuvant therapy (*n* = 69), and conversion therapy (*n* = 36).	Tumor-informed personalized NGS Matched white blood cell DNA was used as a control for variant calling, and plasma variants required confirmation in the matched tumor tissue.	Day 7; 1 month; post-ACT/3–6 months; later serial samples	ctDNA positivity at 7 days and 1 month independently predicted shorter RFS. ACT was associated with improved RFS and OS in ctDNA-positive patients but not ctDNA-negative patients; ctDNA clearance during ACT was associated with better RFS.

Clinical settings: A, chemotherapy-naïve patients undergoing upfront resection for CRLM; B, initially resectable CRLM treated with neoadjuvant systemic therapy; C, initially unresectable CRLM treated with conversion therapy. Mixed cohorts are indicated where applicable. Studies with insufficient information on preoperative treatment or initial resectability were not assigned to a specific category. “Not reported” indicates that the reviewed materials did not explicitly describe the procedure and does not establish that it was not performed. “Not applicable” refers specifically to mutation-based CHIP filtering in assays assessing methylation, fragmentation, or aneuploidy and does not imply absence of hematopoietic background or other sources of false-positive results. Abbreviations: ACT, adjuvant chemotherapy; cfDNA, cell-free DNA; ctDNA, circulating tumor DNA; CHIP, clonal hematopoiesis of indeterminate potential; CNV, copy-number variation; CTC, circulating tumor cell; dPCR/ddPCR, digital/droplet digital PCR; DFS, disease-free survival; EOT, end of treatment; MRD, molecular residual disease; NGS, next-generation sequencing; NR, not reported; OS, overall survival; PFS, progression-free survival; RFS, recurrence-free survival; VAF, variant allele fraction.

## Data Availability

The data analyzed in this review were obtained from the published studies and their Supplementary Materials. The complete data extraction spreadsheet is publicly available on Zenodo (DOI: 10.5281/zenodo.22036664). Study-level hazard ratios, eligibility decisions for quantitative synthesis, risk-of-bias assessments, pooled estimates, and the GRADE assessment are provided in the Supplementary Materials.

## Supplementary Materials

The following supporting information can be downloaded at: https://www.mdpi.com/article/10.3390/cancers18203304/s1, Data S1: Complete database search strategies; Figure S1: Exploratory meta-analysis of postoperative ctDNA positivity and RFS/DFS in two studies using personalized 16-variant NGS assays. The studies used different assay platforms. Unadjusted HRs were pooled using a random-effects model with REML estimation and modified Hartung–Knapp confidence intervals. The wide pooled confidence interval reflects substantial uncertainty; Figure S2: Exploratory meta-analysis of adjusted HRs for the association between early postoperative ctDNA positivity and study-defined RFS/DFS. Estimates were pooled using a random-effects model with REML estimation and modified Hartung–Knapp confidence intervals. Adjustment variables differed between studies. Reporting uncertainties in Reinert et al. and Xu et al. were examined in exclusion sensitivity analyses; Figure S3: Sensitivity analysis restricted to studies with early postoperative sampling before adjuvant chemotherapy supported by explicit text or study protocol diagrams. Unadjusted HRs were pooled using a random-effects model with REML estimation and modified Hartung–Knapp confidence intervals. Protocol diagrams indicate planned sampling and do not confirm the actual timing of sampling relative to treatment in every patient; Table S1: Study-level hazard ratios included in the exploratory meta-analysis; Table S2: Eligibility of the included studies for the exploratory quantitative synthesis; Table S3: Domain-level risk-of-bias assessment of the 28 included studies using the Quality In Prognosis Studies (QUIPS) tool; Table S4: Pooled estimates and heterogeneity statistics for the exploratory meta-analysis and sensitivity analyses; Table S5: GRADE assessment of the association between early postoperative ctDNA positivity and RFS/DFS after curative-intent resection of colorectal liver metastases; Table S6: PRISMA 2020 checklist. Reference [58] is cited in the Supplementary Materials.
