# Supplementary material for: Circulating Tumor DNA in Resected Colorectal Liver Metastases: From Minimal Residual Disease Detection to Treatment Guidance—A Systematic Review
Source: Cancers (Basel). 2026 Oct 14;18(20):3304. doi: 10.3390/cancers18203304 (PMC13702328; doi:10.3390/cancers18203304)
Supplement: Supplementary file 1 [file cancers-18-03304-s001.zip › cancers-4563107-supplementary.pdf]

Circulating Tumor DNA in Resected Colorectal Liver Metastases: From Minimal Residual Disease Detection to Treatment Guidance—A Systematic Review

Melina Yerolatsite, Nanteznta Torounidou, Loizos Hadjigeorgiou, Anna-Lea Amylidi, Evangeli Lampri, Georgios D. Lianos, Angeliki Magklara and George Zarkavelis

Supplementary data

Data S1. Complete database search strategies

Search algorithms

PubMed/MEDLINE

("Colorectal Neoplasms"[Mesh] OR "colorectal cancer"[Title/Abstract] OR "colorectal carcinoma"[Title/Abstract] OR "colorectal neoplasm*"[Title/Abstract] OR "colon cancer"[Title/Abstract] OR "rectal cancer"[Title/Abstract] OR CRC[Title/Abstract]) AND ("liver metastasis"[Title/Abstract] OR "liver metastases"[Title/Abstract] OR "hepatic metastasis"[Title/Abstract] OR "hepatic metastases"[Title/Abstract] OR "colorectal liver metastasis"[Title/Abstract] OR "colorectal liver metastases"[Title/Abstract] OR CRLM[Title/Abstract]) AND ("Circulating Tumor DNA"[Mesh] OR "circulating tumor DNA"[Title/Abstract] OR "circulating tumour DNA"[Title/Abstract] OR ctDNA[Title/Abstract] OR "cell-free DNA"[Title/Abstract] OR "cell free DNA"[Title/Abstract] OR cfDNA[Title/Abstract] OR "liquid biopsy"[Title/Abstract] OR "liquid biopsies"[Title/Abstract] OR "minimal residual disease"[Title/Abstract] OR "molecular residual disease"[Title/Abstract] OR MRD[Title/Abstract])

Scopus

TITLE-ABS-KEY(("colorectal cancer" OR "colorectal carcinoma" OR "colorectal neoplasm" OR "colon cancer" OR "rectal cancer" OR CRC) AND ("liver metastasis" OR "liver metastases" OR "hepatic metastasis" OR "hepatic metastases" OR "colorectal liver metastasis" OR "colorectal liver metastases" OR CRLM) AND ("circulating tumor DNA" OR "circulating tumour DNA" OR ctDNA OR "cell-free DNA" OR "cell free DNA" OR cfDNA OR "liquid biopsy" OR "liquid biopsies" OR "minimal residual disease" OR "molecular residual disease" OR MRD))

Cochrane Library

("colorectal cancer" OR "colorectal carcinoma" OR "colorectal neoplasm" OR "colon cancer" OR "rectal cancer" OR CRC) AND ("liver metastasis" OR "liver metastases" OR "hepatic metastasis" OR "hepatic metastases" OR "colorectal liver metastasis" OR "colorectal liver metastases" OR CRLM) AND ("circulating tumor DNA" OR "circulating tumour DNA" OR ctDNA OR "cell-free DNA" OR "cell free DNA" OR cfDNA OR "liquid biopsy" OR "liquid biopsies" OR "minimal residual disease" OR "molecular residual disease" OR MRD)

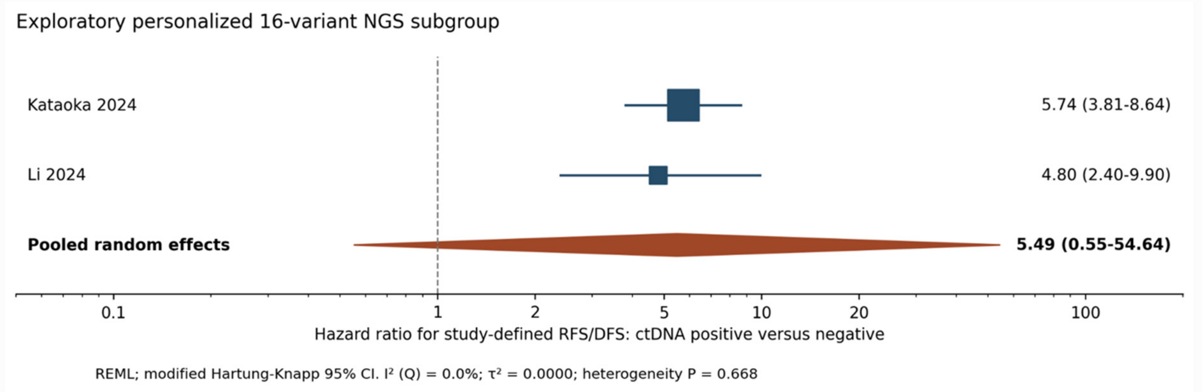

Supplementary Figure S1. Exploratory meta-analysis of postoperative ctDNA positivity and RFS/DFS in two studies using personalized 16-variant NGS assays. The studies used different assay platforms. Unadjusted HRs were pooled using a random-effects model with REML estimation and modified Hartung–Knapp confidence intervals. The wide pooled confidence interval reflects substantial uncertainty.

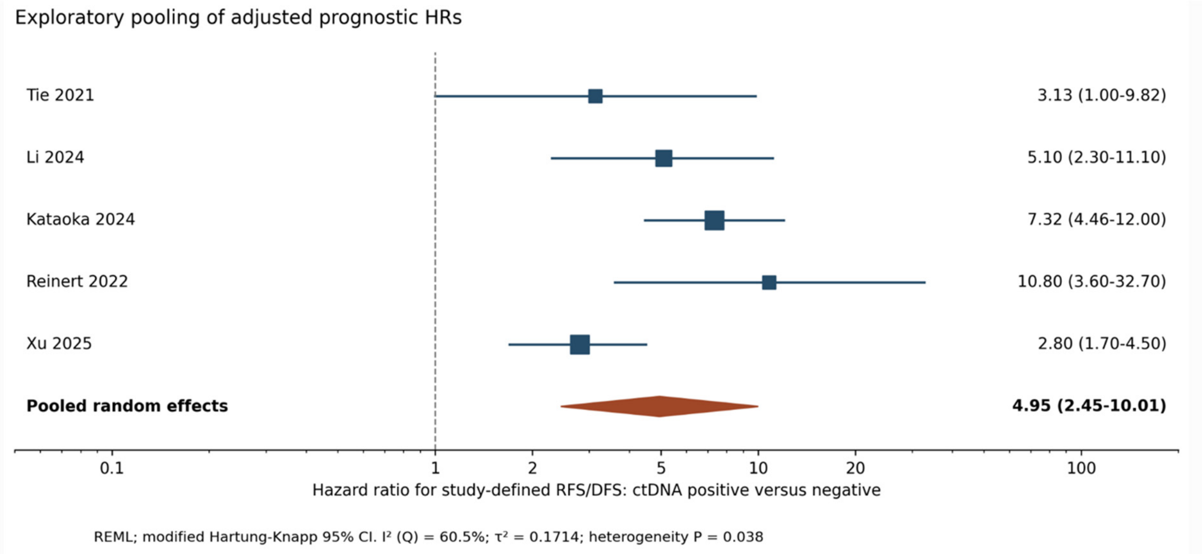

Supplementary Figure S2. Exploratory meta-analysis of adjusted HRs for the association between early postoperative ctDNA positivity and study-defined RFS/DFS. Estimates were pooled using a random-effects model with REML estimation and modified Hartung–Knapp confidence intervals. Adjustment variables differed between studies. Reporting uncertainties in Reinert et al. and Xu et al. were examined in exclusion sensitivity analyses.

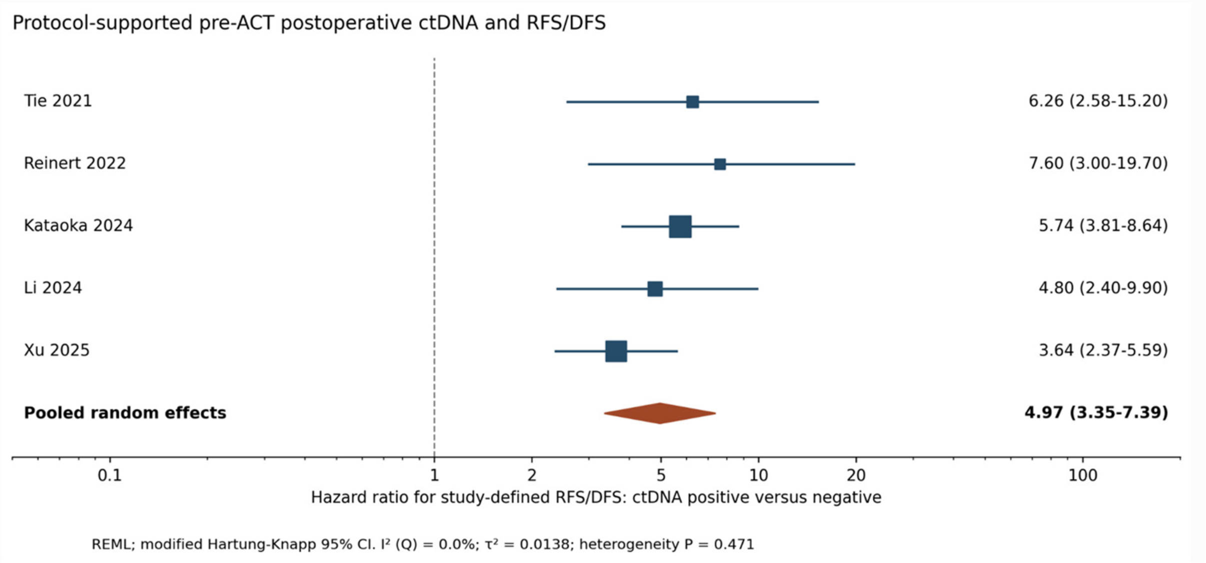

Supplementary Figure S3. Sensitivity analysis restricted to studies with early postoperative sampling before adjuvant chemotherapy supported by explicit text or study protocol diagrams. Unadjusted HRs were pooled using a random-effects model with REML estimation and modified Hartung–Knapp confidence intervals. Protocol diagrams indicate planned sampling and do not confirm the actual timing of sampling relative to treatment in every patient.

Table S1. Study-level hazard ratios included in the exploratory meta-analysis.

A. Unadjusted estimates

Study	Postoperative sampling	Assay	Outcome	Analysis n	HR (95% CI)	Source
**Tie et al., 2021**	4–10 weeks	Tumor-specific single-variant Safe-SeqS	RFS	49	6.26 (2.58–15.20)	Figure 5A
**Reinert et al., 2022**	Day 30 ±14 days	Tumor-informed single-variant ddPCR	RFS	40	7.60 (3.00–19.70)	Figure 2A
**Kataoka et al., 2024**	2–10 weeks	Signatera, personalized 16-plex mPCR-NGS	DFS	189	5.74 (3.81–8.64)	Figure 2A
**Kawashima et al., 2025**	21–30 days	Tumor sequencing followed by patient-specific ddPCR	RFS	53	6.99 (3.19–15.33)	Figure 2A
**Li et al., 2024**	Day 30	Personalized 16-SNV PCR-NGS	RFS	60	4.80 (2.40–9.90)	Figure 2A
**Xu et al., 2025**	1 month	J25 fixed panel with matched-tissue confirmation	RFS	141	3.64 (2.37–5.59)	Figure 2C

B. Adjusted estimates

Study	Model n	HR (95% CI)	Adjustment variables	Source

**Tie et al., 2021**	NR; source subset 49	3.13 (1.00–9.82)	Baseline CEA, liver-metastasis number and size, interval to liver metastases, primary N stage	Table 2
**Reinert et al., 2022**	36	10.80 (3.60–32.70)	Primary N stage	Supplementary Table S8
**Kataoka et al., 2024**	189	7.32 (4.46–12.00)	Prior oxaliplatin exposure, postoperative complications, synchronicity, RAS status, pathological N and T stage, liver-metastasis size and number, primary tumor location, age, sex	Figure 2C
**Li et al., 2024**	NR; source cohort 60	5.10 (2.30–11.10)	Resectability, clinical risk score, preoperative chemotherapy	Table 1
**Xu et al., 2025**	NR; source subset 141	2.80 (1.70–4.50)	Age, primary N stage, liver-metastasis number, bilateral lesions, ACT versus observation	Supplementary Table 4

HRs were interpreted as ctDNA-positive versus ctDNA-negative. The category-order labels in the tables of Tie and Reinert were interpreted using the direction described in the main text and survival figures. Alternative unadjusted table estimates were reported by Tie [6.31 (2.59–15.37)] and Li [4.90 (2.40–9.90)] and were examined in a sensitivity analysis. Pre-ACT sampling was explicitly described in Tie, Reinert, and Kataoka and supported by protocol diagrams in Li and Xu; its timing relative to ACT remained unclear in Kawashima. Protocol diagrams do not verify actual patient-level timing. The exact RFS definition and time origin in Xu were not clearly reported. Source-cohort sizes were not assumed to represent complete-case model sizes when these were not separately reported.

ACT, adjuvant chemotherapy; CEA, carcinoembryonic antigen; CI, confidence interval; ddPCR, droplet digital PCR; DFS, disease-free survival; HR, hazard ratio; mPCR-NGS, multiplex PCR–next-generation sequencing; NR, not reported; RFS, recurrence-free survival; SNV, single-nucleotide variant.

Table S2. Eligibility of the included studies for the exploratory quantitative synthesis.

Study	Included in pooling	Eligibility decision
**Benešová et al., 2019**	No	Early postoperative sampling within one week; no extractable HR for the specified 2–10-week contrast.
**He et al., 2020**	No	Postoperative sampling within seven days, outside the specified window.
**Bolhuis et al., 2021**	No	Sampling ranged from 1 to 99 days; an HR restricted to 2–10 weeks could not be extracted.
**Nishioka et al., 2022**	No	Plasma-only mutation testing and postoperative sampling extending to 180 days.
**Newhook et al., 2023**	No	The reported survival contrast used a tumor-uninformed classifier; no separate eligible HR was available for the tissue-informed subset.
**Chen et al., 2024**	No	Tumor-agnostic methylation assay, outside the selected mutation-based tissue-informed subgroup.
**Raunkilde et al., 2025**	No	Methylation assay, outside the selected assay subgroup.
**Makrodimitris et al., 2026**	No	Methylation-derived tumor fraction, outside the selected assay subgroup.
**Wullaert et al., 2026**	No	Presurgical aneuploidy analysis; no eligible postoperative contrast.
**Moretto et al., 2026**	No	Tumor-naïve assay, outside the selected tissue-informed subgroup.
**Crnovrsanin et al., 2026**	No	Fragmentomic analysis before or during induction therapy; no eligible postoperative contrast.
**Kataoka et al., 2024**	Yes	Personalized multiplex assay; postoperative DFS HRs available.
**Kawashima et al., 2025**	Yes	Patient-specific tissue-informed ddPCR; postoperative RFS HR available.
**Montalti et al., 2025**	No	Tumor-agnostic assay, outside the selected tissue-informed subgroup.
**Narayan et al., 2019**	No	Reported prognostic contrast concerned preoperative plasma mutations.
**Reinert et al., 2022**	Yes	Tissue-informed ddPCR; day-30 RFS HRs available.
**Øgaard et al., 2022**	No	Tumor-agnostic methylation assay; population overlap with Reinert also required consideration.
**Iwai et al., 2020**	No	Non-tumor-specific cfDNA integrity measurement rather than ctDNA MRD detection.
**Jiang et al., 2023**	No	Tumor-naïve mutation panel, outside the selected tissue-informed subgroup.
**Tie et al., 2021**	Yes	Tumor-specific Safe-SeqS assay; postoperative RFS HRs available.
**Wang et al., 2021**	No	Fixed plasma panel; mandatory matching-tumor confirmation of each plasma call was not clearly established in the reported methods.
**Bhangu et al., 2018**	No	Treatment-response analysis; no eligible postoperative RFS/DFS HR.
**Bidard et al., 2019**	No	Baseline, on-treatment, or presurgical ctDNA analysis; no eligible postoperative contrast.
**Polivka et al., 2020**	No	Reported survival contrast concerned preoperative KRAS abundance.
**Kalil et al., 2024**	No	Postoperative sampling window of 1–3 months; an HR restricted to 2–10 weeks could not be extracted.
**Li et al., 2024**	Yes	Personalized 16-variant assay; day-30 RFS HRs available

**Wullaert et al., 2025**	No	Postoperative tracking used variants identified in baseline plasma rather than tumor tissue.
**Xu et al., 2025**	Yes	Fixed J25 panel requiring matched-tissue confirmation; one-month RFS HRs available.

Eligibility decisions apply to the broad exploratory tissue-informed analysis, not to inclusion in the systematic review. Studies excluded from pooling remained in the narrative synthesis. The narrower personalized 16-variant NGS subgroup included only Kataoka and Li. Inclusion of Xu was accompanied by an exclusion sensitivity analysis because the exact RFS definition and time origin were unclear. Reporting uncertainties and sampling details are documented in Table S1. cfDNA, cell-free DNA; ctDNA, circulating tumor DNA; ddPCR, droplet digital PCR; DFS, disease-free survival; HR, hazard ratio; MRD, minimal residual disease; NGS, next-generation sequencing; RFS, recurrence-free survival.

Table S3. Domain-level risk-of-bias assessment of the 28 included studies using the Quality In Prognosis Studies (QUIPS) tool.

Study	D1	D2	D3	D4	D5	D6	Overall
Benešová et al. 2019	M	M	L	H	H	M	High
He et al. 2020	M	M	M	M	H	M	High
Bolhuis et al. 2021	M	M	M	L	M	M	Moderate
Nishioka et al. 2022	H	M	M	L	M	H	High
Newhook et al. 2023	M	M	L	L	M	M	Moderate
Chen, Ji, Xu et al. 2024	M	M	M	L	M	M	Moderate
Raunkilde et al. 2025	M	M	M	L	M	H	High
Makrodimitris et al. 2026	M	M	M	L	M	M	Moderate
Wullaert et al. 2026	L	L	L	L	M	M	Moderate
Moretto et al. 2026	M	M	M	L	M	M	Moderate
Crnovrsanin et al. 2026	M	M	M	L	M	M	Moderate
Kataoka et al. 2024	M	M	L	L	M	M	Moderate
Kawashima et al. 2025	M	M	L	L	H	M	High
Montalti et al. 2025	L	M	M	L	M	M	Moderate
Narayan et al. 2019	M	M	M	L	H	M	High
Reinert et al. 2022	M	M	L	L	M	M	Moderate
Øgaard et al. 2022	M	M	L	L	M	M	Moderate
Iwai et al. 2020†	L	L	L	L	M	M	Moderate
Jiang et al. 2023	M	M	M	L	H	M	High
Tie et al. 2021	M	M	L	L	M	M	Moderate
Wang et al. 2021	M	M	M	L	M	H	High
Bhangu et al. 2018*	M	M	M	M	H	H	High
Bidard et al. 2019	M	M	L	L	M	M	Moderate
Polivka et al. 2020	M	H	M	L	M	H	High
Kalil et al. 2024	M	M	L	L	M	M	Moderate
Li Y, Xu J et al. 2024	M	M	L	M	M	M	Moderate
Wullaert et al. 2025	L	M	M	L	M	M	Moderate

Study	D1	D2	D3	D4	D5	D6	Overall
Xu et al. 2025	M	H	L	M	M	M	High

L, low; M, moderate; H, high risk of bias. D1, study participation; D2, study attrition; D3, prognostic factor measurement; D4, outcome measurement; D5, study confounding; D6, statistical analysis and reporting.

Overall risk was classified as high when at least one domain was high, moderate when at least one domain was moderate and none was high, and low only when all six domains were low. This review-level rule is not a numerical QUIPS score. The assessment yielded 17 moderate-risk studies (60.7%) and 11 high-risk studies (39.3%); none was low risk.

Table S4. Pooled estimates and heterogeneity statistics for the exploratory meta-analysis and sensitivity analyses.

Analysis	Studies, n	Pooled HR (95% CI)	Q (df)	P for heterogeneity	I^2 (%)	τ^2
**Unadjusted: broad tissue-informed analysis**	6	5.15 (3.65–7.27)	4.24 (5)	0.516	0.0	0.0129
**Unadjusted: RFS only**	5	4.98 (3.08–8.05)	3.74 (4)	0.443	0.0	0.0262
**Unadjusted: personalized 16-variant NGS assays**	2	5.49 (0.55–54.64)	0.18 (1)	0.668	0.0	0.0000
**Unadjusted: pre-ACT sampling explicitly described in the text**	3	6.04 (2.83–12.90)	0.29 (2)	0.863	0.0	0.0000
**Unadjusted: pre-ACT sampling supported by text or protocol diagrams**	5	4.97 (3.35–7.39)	3.55 (4)	0.471	0.0	0.0138
**Unadjusted: excluding Xu et al.**	5	5.93 (3.94–8.93)	0.82 (4)	0.936	0.0	0.0000
**Unadjusted: alternative table estimates for Tie and Li**	6	5.17 (3.66–7.30)	4.24 (5)	0.516	0.0	0.0132
**Unadjusted: DerSimonian–Laird sensitivity analysis**	6	5.09 (3.72–6.97)	4.24 (5)	0.516	0.0	0.0000
**Adjusted: all available estimates**	5	4.95 (2.45–10.01)	10.14 (4)	0.038	60.5	0.1714
**Adjusted: excluding Reinert et al.**	4	4.41 (1.90–10.20)	7.85 (3)	0.049	61.8	0.1550
**Adjusted: excluding Xu et al.**	4	6.46 (3.54–11.78)	2.97 (3)	0.396	0.0	0.0000
**Adjusted: excluding Reinert and Xu**	3	5.99 (2.43–14.75)	2.03 (2)	0.363	1.4	0.0078

Supplementary Table S5. GRADE assessment of the association between early postoperative ctDNA positivity and RFS/DFS after curative-intent resection of colorectal liver metastases.

Scope: tumor-informed assays, one early postoperative estimate per cohort, and ctDNA-positive versus ctDNA-negative patients. The rating concerns whether an adverse prognostic association is present (HR >1), rather than a precise common HR, an individual absolute recurrence risk, or the benefit of ctDNA-guided management. This assessment was added during manuscript revision.

**Evidence profile**	**Unadjusted association**	**Adjusted association**
Evidence base	6 cohorts; 532 patients	5 cohorts; complete-case model N incompletely reported
Pooled HR (95% CI)	5.15 (3.65–7.27)	4.95 (2.45–10.01)
Statistical heterogeneity	I^2 = 0%; τ^2 = 0.0129	I^2 = 60.5%; τ^2 = 0.1714
Starting certainty	High[1]	High[1]
Risk of bias	Serious (−1)[2]	Serious (−1)[2]
Inconsistency	Not serious for direction[3]	Not serious for direction; magnitude varies[3]
Indirectness	Not serious for specified question[4]	Not serious for specified question[4]
Imprecision	Not serious for HR >1[5]	Not serious for HR >1[5]
Publication bias	Uncertain; no additional downgrade[6]	Uncertain; no additional downgrade[6]
Upgrading	None[7]	None[7]
**Overall certainty**	**MODERATE**	**MODERATE**

Studies: Tie 2021, Reinert 2022, Kataoka 2024, Kawashima 2025, Li 2024 and Xu 2025. The adjusted synthesis excludes Kawashima because its multivariable early-recurrence analysis is not an adjusted RFS HR. These are overlapping analyses of the same evidence base, not independent replications.

CI, confidence interval; ctDNA, circulating tumor DNA; DFS, disease-free survival; GRADE, Grading of Recommendations Assessment, Development and Evaluation; HR, hazard ratio; RFS, recurrence-free survival.
